# Superconducting phase transitions in disordered NbTiN films

**DOI:** 10.1038/s41598-020-58192-3

**Published:** 2020-01-30

**Authors:** M. V. Burdastyh, S. V. Postolova, T. Proslier, S. S. Ustavshikov, A. V. Antonov, V. M. Vinokur, A. Yu. Mironov

**Affiliations:** 1 A. V. Rzhanov Institute of Semiconductor Physics SB RAS, 13 Lavrentjev Avenue, Novosibirsk, 630090 Russia; 2 Novosibirsk State University, Pirogova str. 2, Novosibirsk, 630090 Russia; 3 Institute for Physics of Microstructures RAS, GSP-105, Nizhny Novgorod, 603950 Russia; 4 Institut de recherches sur les lois fundamentales de l’univers, Commissariat de l’énergie atomique et aux énergies renouvelables-Saclay, Gif-sur-Yvette, France; 5 Materials Science Division, Argonne National Laboratory, 9700 S. Cass Ave, Argonne, IL 60439 USA

**Keywords:** Surfaces, interfaces and thin films, Superconducting properties and materials

## Abstract

Suppression of superconductivity in disordered systems is a fundamental problem of condensed matter physics. Here we investigate superconducting niobium-titanium-nitride (Nb_1−*x*_Ti_*x*_N) thin films grown by the atomic layer deposition (ALD) with slightly different growth process parameters. We observe a smooth crossover from the disorder-driven superconductor-normal metal transition (SMT) to the superconductor-insulator transition (SIT) via the intermediate Bose metal state detected by the low-temperature saturation of the temperature dependence of the sheet resistance. We demonstrate that the SIT via the intervening Bose metal state occurs if the sheet resistance of the film in the maximum, *R*_max_ prior to the superconducting drop of *R*(*T*), exceeds *R*_*q*_ = *h*/4*e*^2^.

## Introduction

The superconductor-insulator transitions observed in a wealth of various systems^1,2^, are often grouped into two distinct categories depending on the presumed mechanism of superconductivity suppression — fermionic^3^ or bosonic^4,5^. The fermionic scenario assumes that disorder destroys the Cooper pairs, and that the further increase of disorder leads to localization of normal carriers and formation of the insulating state. In this case, as the normal state sheet resistance ${R}_{\square N}$, which serves as a measure of disorder, increases, both superconducting critical temperature ${T}_{c}$ at which the fluctuating Cooper pairs acquire an infinite life-time and the Berezinskii-Kosterlitz-Thouless (BKT) transition temperature, ${T}_{BKT}$, at which the global phase coherence establishes, reduce down towards zero temperature. Then the superconductor-normal metal transition (SMT) occurs due to the complete disappearance of the Cooper pairs, and the suppression of ${T}_{c}$ with ${R}_{\square N}$ growth is well described by the Finkel’stein formula^3^. Such a behaviour is observed, for example, in thin films of niobium nitride NbN^6,7^. In the bosonic scenario, increasing disorder localizes Cooper pairs, which survive across the SIT and maintain in the insulating state. Accordingly, as ${R}_{\square N}$ increases, ${T}_{c}$ decreases weakly and ${T}_{BKT}$ is suppressed down to zero at the SIT. In this scenario the SIT occurs across a single point of the metallic phase with the critical sheet resistance ${R}_{q}=h$/$4{e}^{2}=6.45$ k$\Omega$^5^. Experimentally, the critical sheet resistance at the SIT is not universal and varies in the range between approximately ${R}_{q}$/2 and $3{R}_{q}$. The direct disorder-driven SIT is observed, for example, in indium oxide InO${}_{x}$^8–10^ and titanium nitride TiN^11,12^ films. The possible intermixing of two scenarios was observed, for example in^13^. The review of the materials manifesting the SIT via the Bose metal state is given in^14,15^, see also^16^. Until recently, the interrelation between these mechanisms and especially the nature of the intermediate Bose metal^17–22^ have been remaining not completely understood and have been intensively debated^2,15,23^. It is proposed in^16^ that the intermediate Bose metal is a bosonic topological insulator with its conductance provided by topologically protected edge Cooper pair states.

In what follows we report our measurements of the disorder-driven SIT taken on the three sets of Nb${}_{1-x}$Ti${}_{x}$N films grown by ALD technique. The difference between the sets is achieved by the slight variation of ALD parameters, the fraction $x$ and the temperature of deposition ${T}_{ALD}$. We demonstrate that with the increasing $x$ and decreasing ${T}_{ALD}$, a smooth crossover from the SMT to the SIT occurs. We show that the intervening Bose metal emerges if the sheet resistance of the film at the maximum, ${R}_{\max }$, which the ${R}_{\square }(T)$ dependence achieves prior to falling to the superconducting state, exceeds ${R}_{q}=h$/$4{e}^{2}$. We reveal that the perpendicular magnetic field turns Bose metal into an insulator.

## Samples Preparation and Characterization

We grow NbTiN films, by employing the atomic layer deposition (ALD) technique based on the sequential surface reaction step-by-step film growth. This highly controllable process provides superior thickness and stoichiometric uniformity and an atomically smooth surface^24,25^ as compared to chemical vapor deposition, the standard technique used to grow NbTiN films^26^. We used NbCl${}_{5}$, TiCl${}_{4}$, and NH${}_{3}$ as gaseous reactants; the stoichiometry was tuned by varying the ratio of TiCl${}_{4}$/NbCl${}_{5}$ cycles during growth^27^. The superconducting properties of these ultrathin NbTiN films were optimized by utilizing AlN buffer layers grown on top of the Si substrate^28^. All films have a fine-dispersed polycrystalline structure^29^ with the average crystallite size being $\approx 5$ nm.

Three sets of Nb${}_{1-x}$Ti${}_{x}$N films are grown varying deposition temperature ${T}_{ALD}$ and fraction of Ti $x$. For Set-1 ${T}_{ALD}\,=$ 450 ${}^{\circ }$C, and ${T}_{ALD}\,=$ 350 ${}^{\circ }$C for Set-2 and Set-3. The Ti fraction $x=0.3$ in Set-1 and Set-2 and $x=0.33$ in Set-3. Films within single Set are grown varying the number of ALD cycles, that provides films of different thickness $d$. The parameters of samples are given in the Table 1. The Hall carrier density $n$ (see SI) appears to be approximately the same regardless of the disorder $n \sim 1{0}^{22}$ cm${}^{-3}$ which is one order smaller that in NbTiN films examined in^30^.

**Table 1:** ${T}_{ALD}$ is the deposition temperature; $d$ is film thickness; ${R}_{\max }$ is the resistance at the maximum of $R(T)$; ${R}_{77}$ is the resistance per square at $T=77$ K; ${T}_{c}$ is the critical temperature determined from the SF-fits; ${T}_{BKT}$ is BKT transition temperature; $D$ is the diffusion coefficient $D=0.882\cdot {T}_{c}$/$(e{B}_{c2})$ (see Fig. 2 in SI for ${B}_{c2}$); $n$ is the Hall carrier density (see SI). In all investigated Nb_1−x_Ti_x_N films the mean free path is very small (same order as lattice constant), so all samples are in dirty limit $l < {\xi }_{0}$. Films S3-1, S3-2 and S3-3 are same films as S3-4, but that have degraded with time.

Sample Nb${}_{1-x}$Ti${}_{x}$N		${\boldsymbol{d}}$ (nm)	${{\boldsymbol{R}}}_{{\bf{\max }}}$ (k$\Omega$)	${{\boldsymbol{R}}}_{{\bf{77}}}$ (k${\boldsymbol{\Omega }}$)	${{\boldsymbol{T}}}_{{\bf{c}}}$ (K)	${{\boldsymbol{T}}}_{{\bf{BKT}}}$ (K)	D $\frac{{{\bf{cm}}}^{{\bf{2}}}}{{\boldsymbol{s}}}$	n $\frac{{{\bf{10}}}^{{\bf{22}}}}{{{\bf{cm}}}^{{\bf{3}}}}$
Set-1, *x* = 0.3 *T*_*ALD*_ = 450 °C	S1-1	3	3.96	2.56	0	0	—	—
	S1-2	10	0.75	0.69	4.85 ± 0.005	4.79 ± 0.005	—	—
	S1-4	20	0.18	0.17	6.26 ± 0.005	6.11 ± 0.005		
Set-2, *x* = 0.3 *T*_*ALD*_ = 350 °C	S2-1	10	17.55	4.52	0	0	—	0.5
	S2-2	12	5.65	2.85	2 ± 0.005	1.75 ± 0.005	—	—
	S2-3	15	2.66	1.69	3.27 ± 0.005	3.08 ± 0.005	0.2	—
	S2-5	40	0.52	0.77	4.33 ± 0.005	4.18 ± 0.005	—	—
Set-3, *x* = 0.33 *T*_*ALD*_ = 350 °C	S3-1	9.2	17.9	—	—	0	—	—
	S3-2	9.2	15.72	—	—	0	—	—
	S3-3	9.2	15.18	5.75	0.75 ± 0.005	0	—	—
	S3-4	9.2	14.13	—	0.97 ± 0.005	0	—	—
	S3-5	10	9.26	4.53	1.7 ± 0.005	0	—	—
	S3-7	12	2.24	1.87	3.85 ± 0.005	3.81 ± 0.005	—	—
	S3-9	19	1.87	0.98	4.28 ± 0.005	4.26 ± 0.005	0.3	1
	S3-10	21	0.8	0.69	4.35 ± 0.005	4.29 ± 0.005	—	—

## Results and Discussion

**Figure 1:**
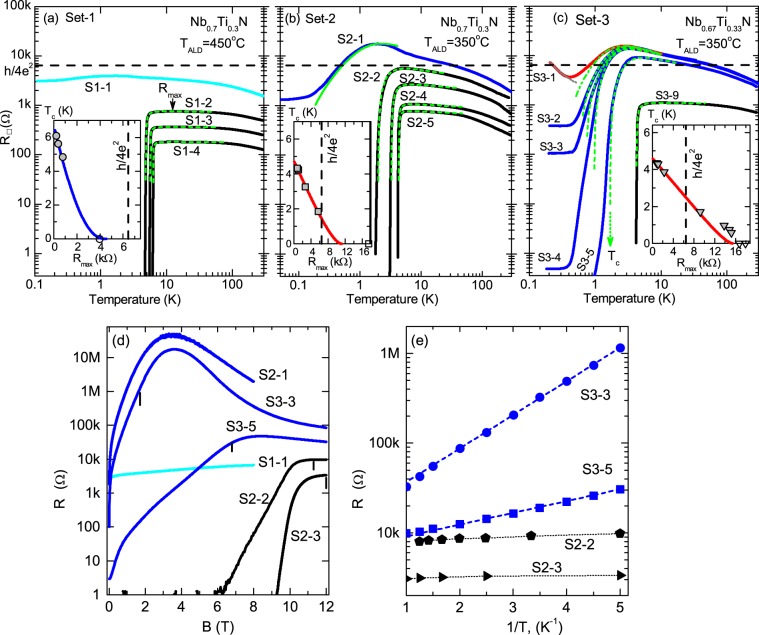
Sheet resistance ${R}_{\square }$ vs. temperature on the log-log scale for films of Set-1 (**a**), Set-2 (**b**) and Set-3 (**c**). The deposition temperature ${T}_{ALD}$ and Ti fraction $x$ in Nb${}_{1-x}$Ti${}_{x}$N composition are given on the plots (see SI for the line-log scale). The vertical axis scale is same for all plots. Solid lines are experimental dependencies, where blue lines show Bose metal samples. Horizontal dashed lines shows the resistance ${R}_{q}=h$/$4{e}^{2}=6,45$ k$\Omega$. Dashed green lines: fits accounting for contributions to conductance from superconducting fluctuations (SF), the ${T}_{c}$ obtained from these fits are given in Table. (**c**) Gray dotted lines: activation dependence ${R}_{\square }\propto \exp (1/T)$ for samples S3-0 and S3-1. Sample S3-5 doesn’t manifest BKT transition (see Fig. 2(b,d)). Insets: ${T}_{c}$ vs. sheet resistance in maximum ${R}_{\max }$ prior to superconducting resistance drop, symbols are the experimental values, the solid line is the theoretical fitting by Eq. (1) with the adjustable parameter $\gamma =$6.5 (${T}_{c0}=6.71$ K) for (**a**); $\gamma \,=$ 4.4 (${T}_{c0}=4.75$ K) for (**b**); and $\gamma \,=$ 3.8 (${T}_{c0}=4.6$ K) for (**c**). Dashed vertical lines shows the ${R}_{q}$. Open symbols with ${T}_{c}=0$ correspond to samples for which we can not reliably define ${T}_{c}$ with SF-fits. (**d**) Magnetoresistance per square ${R}_{\square }(B)$ on semi-log scale for films listed in figure. All curves are taken at temperature $T=0.2$ K, except for ${R}_{\square }(B)$ of sample S1-1 that is obtained at $T=0.04$ K. Vertical black segments mark the magnetic fields in which $R(1/T)$ in (**e**) were obtained. (**e**) Arrhenius plot of sheet resistance ${R}_{\square }$ in constant perpendicular magnetic field vs. 1/$T$ for samples S3-3 ($B$=1.7 T), S3-5 ($B$ = 6.8 T), S2-2 ($B=11.3$ T) and S2-3 ($B=12$ T). Dashed lines show the activation dependence $R={R}_{I}\exp ({E}_{I}/{k}_{B}T)$, where for S3-3 ${R}_{I}$ = 5.3 k$\Omega$, ${E}_{I}$ = 86 meV and for S3-5 ${R}_{I}$ =2.3 k$\Omega$, ${E}_{I}$ =26 meV. Samples S2-2 and S2-3 exhibit saturation (not activation).

 Figure 1 presents the temperature dependencies of the sheet resistance ${R}_{\square }(T)$ for three our Sets of films (Fig. 1(a–c)) together with magnetoresistance curves $R(B)$ (Fig. 1(d–f)) for samples with ${R}_{\max }$ being close to ${R}_{q}=h$/$4{e}^{2}$. A qualitative difference between Set-1 and Sets-2,3 is that the superconductivity in Set-1 (Fig. 1(a)) gets fully suppressed, with the sheet resistance of samples in maximum ${R}_{\max }$ increasing, before ${R}_{\max }$ reaches ${R}_{q}$. In Set-3 (and most likely in Set-2), samples with ${R}_{\max } > {R}_{q}$ still experience superconducting transition, and Sets-2,3 demonstrate more complicated evolution.

Taking into account quantum contributions to conductivity from superconducting fluctuations (SF) at $T > {T}_{c}$ and weak localization^31–36^ (see SI for details), we fit the experimental data (dashed green lines in Fig. 1(a–c)). These SF-fits, in which the critical temperature ${T}_{c}$ enters as the adjustable parameter, yield the macroscopic value of ${T}_{c}$. For all samples, the extracted ${T}_{c}$ (given in the Table) is very close to the temperature of the inflection point, i.e. the temperature where $dR$/$dT$ is maximal^37^, and lies at the foot of ${R}_{\square }(T)$^38^.

### Metal with Cooper pairing

 Figure 1(a–c) demonstrate that the SF-fitting describes fairly well the graduate decrease in the resistance ${R}_{\square }(T)$ matching perfectly the experimental points (without any additional assumptions about mesoscopic inhomogeneities^39^) for all superconducting samples of Set-1 and the most part of samples from Set-2 and Set-3. The macroscopic superconductivity in samples of Set-1 is fully suppressed before sample’s ${R}_{\max }$ reaches ${R}_{q}$. Figure 1(d) shows ${R}_{\square }(B)$ curve of non-superconducting sample S1-1 taken at $T=0.04$ K. The resistance depends weakly on magnetic field (without a magnetoresistance peak presented in samples with close ${R}_{\max }$ value from other Sets), implying the lack of Cooper pairs in the sample.

Samples with ${R}_{\max } > {R}_{q}$, i.e. the samples S2-1, S3-5, S3-4, S3-3 and S3-2 show the significant resistance drop (from few orders of magnitude for sample S3-5 to one order for S3-1). The appreciable parts of these drops exhibit a perfect match with the SF-fits, implying that there are short-living Cooper pairs in a system. But the global coherent superconducting state is not achieved even at lowest temperatures. After deviating from the SF-fits, the ${R}_{\square }(T)$ either increases exponentially (S3-1, S3-0) evidencing that the system transforms into an insulating state (thinner films, not shown in Fig. 1(c), are insulators^40^) or saturates (samples from S3-5 to S3-2), evidencing these films fall into the Bose metallic state, harboring a finite density of free vortices. Note that already the early study of the ultrathin amorphous Ga films have demonstrated^41^ the role of the relation between the normal state sheet-resistance (taken at 14 K) and the quantum resistance, in determining whether the film falls into a superconductor or saturates at lower temperatures to some metallic state.

Magnetic field reveals a huge difference between ${R}_{\square }(B)$ corresponding to the samples S2-1, S3-5, S3-4, S3-3 and S3-2 (${R}_{\max } > {R}_{q}$) exhibiting the Bose metal and samples S2-2, S2-3 (${R}_{\max } < {R}_{q}$) (Fig. 1(d)). The magnetoresistance of samples with ${R}_{\max } < {R}_{q}$ appears only in strong magnetic field (we do not see maximum for S2-2 and S2-3 since for superconducting samples it appears at higher fields^12^). The magnetoresistance of Bose metal films shoots up with slight increase of magnetic field from zero, then ${R}_{\square }(B)$ reaches a maximum, followed by decrease of ${R}_{\square }(B)$. Both observed features, the magnetoresistance (MR) peak and the fast increase of ${R}_{\square }(B)$ in weak magnetic field, become more pronounced with the increasing ${R}_{\max }(B=0)$.

 Figure 1(e) highlights the crucial feature of the ${R}_{\square }(T,B=const)$ in constant magnetic field for Bose metal samples. Samples with ${R}_{\max }(B=0) < {R}_{q}$ show almost flat $R(T)$ dependence in magnetic field, while the samples with ${R}_{\max }(B=0) > {R}_{q}$ turn into insulator with typical insulating dependence $R={R}_{I}\exp ({E}_{I}/{k}_{B}T)$. The difference is most remarkable for samples S2-2 and S3-5 — $R(T)$ for these samples are quite similar, but the behaviour in magnetic field is qualitatively different. As was shown in details in^29^, in constant magnetic field sample S2-1 demonstrates the temperature-driven charge BKT transition into superinsulating state^29,42^ — the state also found in the InO films^43,44^. The giant MR peak and Arrhenius behavior of the resistance near it may be found in systems in which there are large fluctuations in the amplitude of the superconducting order parameter^2^. Experimentally these fluctuation appear to be helped by compositional variations on a mesoscopic scale^2^. Apparently, the mesoscopic compositional variations emerge in our Sets with decreasing deposition temperature ${T}_{ALD}$ and increasing fraction of Ti $x$.

To sum up, we observe that the interrelation between superconducting film’s sheet resistance in the maximum, ${R}_{\max }$, and ${R}_{q}=h$/$4{e}^{2}$ is the criteria of the Bose metal appearance. We demonstrate that in magnetic field Bose metal turns into insulator with the Arrhenius $R\propto \exp (1/{k}_{B}T)$ behaviour.

### Suppression of superconductivity

Now we discuss the possible scenarios of suppression of the superconductivity in our system. The transition into a superconducting state in thin films is a two-stage process. First, the finite amplitude of the order parameter forms at the superconducting critical temperature ${T}_{c}$, second, a global phase-coherent state establishes at lower temperature, ${T}_{BKT}$, of the Berezinskii-Kosterlitz-Thouless (BKT) transition. Below we analyze how both these temperatures reduce with growing disorder.

The suppression of ${T}_{c}$ with the increase of sample’s ‘normal’ sheet resistance (insets in Fig. 1). In fermionic scenario, the suppression of ${T}_{c}$ follows celebrated Finkelstein’s formula^3^:

1 $$ln\left(\frac{{T}_{c}}{{T}_{c0}}\right)=\frac{1}{| \gamma | }-\frac{1}{\sqrt{2r}}ln\left(\frac{\gamma -r/4-\sqrt{r/2}}{\gamma -r/4+\sqrt{r/2}}\right),$$

where $\gamma =1$/$ln(k{T}_{c0}\tau /\hslash )$ (${T}_{c0}$ is superconducting critical temperature of a clean sample) and $r={G}_{00}{R}_{{\square }_{N}}$, where the choice of ‘normal state’ sheet resistance is uncertain due to strong non-monotonic ${R}_{\square }(T)$ dependence. Usually, to analyze the suppression of ${T}_{c}$ with disorder, ${T}_{c}$ is plotted vs. resistance at some temperature^45,46^. For our samples we plot ${T}_{c}$ vs. ${R}_{\max }$, the resistance in the maximum prior to superconducting resistance drop.

**Figure 2:**
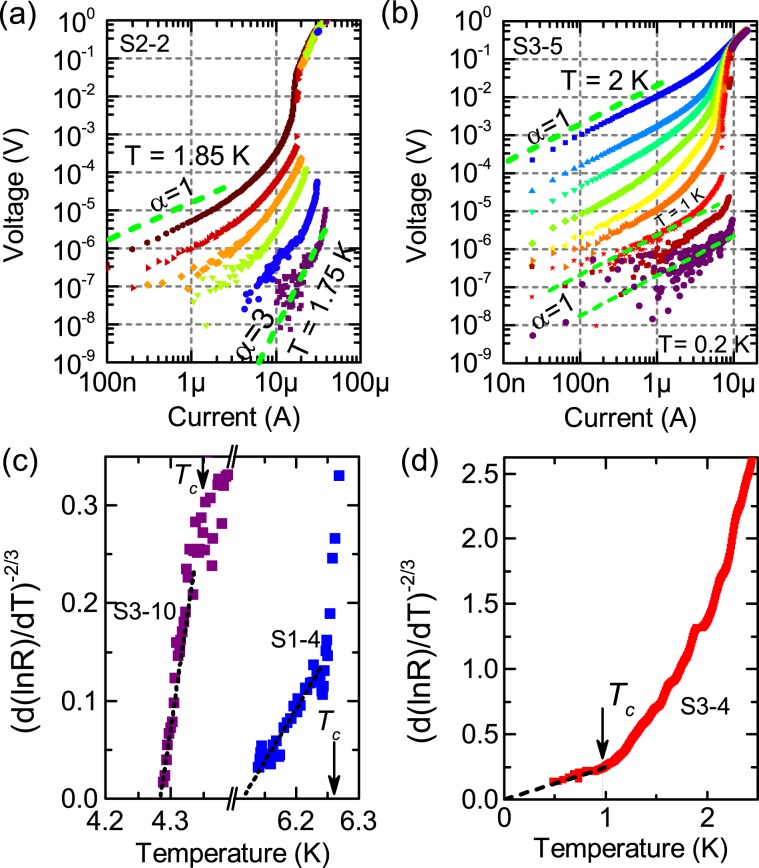
(**a**,**b**) Temperature evolution of current-voltage characteristics on a log-log scale for samples S2-2 and S3-5. Dashed line indicate the slopes corresponding to power $\alpha =1$ and $\alpha =3$ on the $V\propto {I}^{\alpha }$. (**c**), (**d**) Rescaling of the sheet resistance ${R}_{\square }(T)$ to the BKT form to extract the vortex-unbinding temperature ${T}_{BKT}$ (Eq. (2)) for samples S1-4, S3-10 (**c**) and sample S3-4 (**d**), where the straight line (dashed) corresponds to Eq. (2). Arrows mark position of ${T}_{c}$. Notably, the ${R}_{\square }(T)$ curves obey Eq. (2) just at $T < {T}_{c}$. The value ${(d(lnR)/dT)}^{-2/3}=0$ at ${T}_{BKT}$.

The hallmark manifestation of the BKT transition are the critical behavior of the resistance above the transition

2 $$R(T)\propto \exp [const/{(T/{T}_{BKT}-1)}^{1/2}].$$

and the power-law behaviour of current-voltage characteristics $V\propto {I}^{\alpha \ge 3}$ at $T < {T}_{BKT}$. Then the transition temperature can be found as a temperature at which $\alpha$ experiences a jump 1$\to$3 at ${T}_{BKT}$ The second method is to replot $R(T)$ (see Eq. (2)) as ${(d(lnR)/dT)}^{-2/3}(T)$^47^. If the decay of resistance is due to vortex motion the experimental curve is linear in these coordinates and the intersection of experimental curve with axis $x$ corresponds to ${T}_{BKT}$ (Figs. 2(c,d) and 5 in SI). As was demonstrated for ALD deposited TiN films^36^, these methods provide BKT temperatures that coincide within one percent. Below we utilize both methods to observe BKT transition or it’s absence in a system.

The procedure of fitting the resistance with the particular theory is as follows. First, taking quantum contributions to conductivity from superconducting fluctuations (SF) at $T > {T}_{c}$ and weak localization^31–36^ (see SI for details), we fit the experimental $R(T)$ in the region around $R(T)$ reaching ${R}_{\max }$ and further decreasing with cooling (dashed green lines in Fig. 1). These SF-fits, in which critical temperature ${T}_{c}$ enters as the adjustable parameter, yield the macroscopic value of ${T}_{c}$. For all samples, the extracted ${T}_{c}$ (given in Table) is very close to the temperature of the inflection point, i.e. the temperature where $dR$/$dT$ is maximal^37^, and lies at the foot of ${R}_{\square }(T)$^38^. The $R(T)$ deviates from SF-fit below the obtained ${T}_{c}$, so we fit the region $T < {T}_{c}$ with Eq. (2), but before doing that we analyze the current-voltage dependencies to define ${T}_{BKT}$ (Fig. 2(a,b)).

In Set-1 the values of ${T}_{c}$ plotted vs. ${R}_{\max }$ (inset of Fig. 1(a)) show that the suppression of ${T}_{c}$ is in accord with Eq. (1), with fitting parameter $\gamma \,=$       6.5. Below ${T}_{c}$ the ${R}_{\square }(T)$ follows Eq. (2) (see Figs. 2(c) and 5 in SI) and, hence, is caused by motion of free vortices. The obtained ${T}_{BKT}$ temperatures are listed in the Table. Keeping in mind that the resistance depends weakly on the magnetic field (Fig. 1(d)), i.e. the lack of Cooper pairs in the sample, we conclude that the Set-1 exhibits the SMT in agreement with fermionic scenario.

We observe in Set-2 that both the power-law $V(I)$ curves (Fig. 2(a)) and $R(T)$ dependencies at $T < {T}_{c}$ (see Fig. 5 in SI) are in agreement with standard BKT theory without any additional assumptions about effect of disorder^39^ even for sample S2-2 that has ${R}_{\max }\lesssim {R}_{q}$. The suppression of ${T}_{c}$ in Set-2 (inset in Fig. 1(b)) is described with the fitting parameter $\gamma \,=$ 4.4. That is slightly below the applicability limit of Eq. (1) which is $\gamma \simeq 5$. Unfortunately, we do not have samples between S2-1 and S2-2 so we can not tell, how ${T}_{c}$ gets suppressed in region ${R}_{\max } > {R}_{q}$. Since the behavior of S2-1 shows (Fig. 1(d)) that the film falls into metallic state with Cooper pairing, the behaviour of Set-2 suggests the action of both fermionic and bosonic mechanisms of superconductivity suppression.

The films of Set-3 fall into two categories depending on the ratio between ${R}_{\max }$ and ${R}_{q}$ (Fig. 1(c)). For films with ${R}_{\max } < {R}_{q}$ the ${R}_{\square }(T)$ decrease with cooling down is in agreement with the conventional theories of superconducting fluctuations and Berezinskii-Kosterlitz-Thouless transition (Fig. 2(c)), where both ${T}_{BKT}$ and ${T}_{c}$ decrease with ${R}_{\max }$ increasing (see Table).

The transition temperature ${T}_{c}$ obtained from the SF-fits decreases with ${R}_{\max }$ increasing (inset in Fig. 1(c)). Equation (1) describes the ${T}_{c}$ suppression but only for samples with ${R}_{\max } < {R}_{q}$ and with quite small $\gamma =$3.8, when normally $\gamma \gtrsim 5$ in fermionic model. For samples with ${R}_{\max } > {R}_{q}$ the critical temperature ${T}_{c}$ decreases slower than Eq. (1) predicts.

Bose metal films (${R}_{\max } > {R}_{q}$) do not experience the BKT transition. Particularly, for S3-5 and S3-4 the $R(T)$ demonstrates decay in accord with Eq. (2) but yields ${T}_{BKT}=0$ (Fig. 2(d)) and $V(I)$ curves in low-current limit remain linear $V\propto {I}^{\alpha =1}$ at all measured temperatures (Fig. 2(b)), only the jump in $V(I)$ develops slightly below ${T}_{c}$. The absence of the classical BKT behaviour could be due to inhomogeneities of samples, but if we estimate the scale of inhomogeneities $L=4k{T}_{BKT}$/${\Phi }_{0}* {L}_{phys}/{I}^{* }$^48^ where as ${T}_{BKT}$ we take temperature at which the jump in $V(I)$ appears, ${L}_{phys}=50$  $\mu$m is physical size of sample and ${I}^{* }\simeq 3$ $\mu$A is the current above which current-induced free vortices lead to the power-law dependence^48^, we obtain the value of order $L\simeq 1$ mkm. This is few orders of magnitude larger that inhomogeneities observed from electronic and atomic-force microscopy. Even though the exponent $\alpha$ remain $\alpha =1$, the voltage jumps to normal resistance branch with increasing $I$ appear at low temperatures. In disordered ALD deposited TiN films, these voltage jumps in $V(I)$ occur at $T\simeq {T}_{BKT}$, i.e. when $\alpha$ switches from 1 to 3^49^. Hence, we observe that the ${T}_{BKT}$ transition vanishes while ${T}_{c}$ remains non-zero. Hence, for superconducting films with ${R}_{\max } > {R}_{q}$, the ${T}_{BKT}$ transition vanishes while ${T}_{c}$ remains non-zero. This is typical for bosonic scenario of suppression of superconductivity^4,5^.

## Conclusion

We have examined three sets of superconducting disordered thin Nb${}_{1-x}$Ti${}_{x}$N films, where the only difference between sets was the fraction of Ti $x$ and/or the temperature of deposition ${T}_{ALD}$. We showed that, both increase of $x$ and the decrease of ${T}_{ALD}$, lead to the smooth crossover from fermionic mechanism of superconductivity suppression to the case where both bosonic and fermionic mechanisms are involved. We show that the ratio between ${R}_{\max }$, and ${R}_{q}=h$/$4{e}^{2}$ divides films of all sets into two categories. For moderately disordered films (${R}_{\max } < {R}_{q}$) the superconducting transition is in agreement with the conventional theories of superconducting fluctuations and Berezinskii-Kosterlitz-Thouless transition, and ${T}_{c}$ decreases with disorder in accord with the Finkel’stein formula. For critically disordered films (${R}_{\max } > {R}_{q}$) the ${T}_{c}$ decreases slower then the Finkel’stein formula predicts. Moreover, films with ${R}_{\max } > {R}_{q}$ do not experience the BKT transition to zero-resistance state. Careful magnetoresistance measurements revealed that there is a qualitative difference between films with ${R}_{\max }$ smaller and greater than ${R}_{q}$.

## Methods

The fabrication is built upon the Atomic Layer Deposition technique. The structure of films grown on Si substrates with AlN buffer layers was investigated using a JEOL-4000EX electron microscope operated at 400 kV, with a point-to-point resolution of 0.16nm and a line resolution of 0.1 nm.

### Measurement technique

The films were lithographically patterned into bridges 50 $$\mu $$m wide, the distance between current-contacts was 2500 $$\mu $$m and distance between voltage-contacts was 450 $$\mu $$m. Low resistive transport measurements ($$R(B,T) < 1$$ M$$\Omega $$) are carried out using low-frequency ac and dc techniques in a four-probe configuration $$I=1-10$$ nA, $$f\approx 3$$ Hz. High resistive transport measurements ($$R(B,T) > 1$$ M$$\Omega $$) are carried out using low-frequency ac and dc techniques in a two-probe configuration with $$V\approx 100$$$$\mu $$V, $$f\approx 1$$ Hz. For ac measurements we use one/two SR830 Lock-ins and current/voltage preamplifiers SR570/SR560. For dc measurements we use sub-femtoampermeter Keythley 6430a and nanovoltmeter Agilent 34420. All resistance measurement are carried out in linear regime with using adequately system of filtration. Resistivity measurements at sub-Kelvin temperatures were performed in dilution refrigerators $${}^{3}$$He/$${}^{4}$$He with superconducting magnet.

## Supplementary information

Supplementary Information

## Data Availability

The authors declare that all relevant data supporting the findings of this study are available within the article and its supplementary information file. Additional raw data, if necessary, are available upon request to AYuM, mironov@isp.nsc.ru
