# Supplementary material for: Superconducting phase transitions in disordered NbTiN films
Source: Sci Rep. 2020 Jan 30;10:1471. doi: 10.1038/s41598-020-58192-3 (PMC6992621; doi:10.1038/s41598-020-58192-3)
Supplement: Supplementary file 1 — Supplementary Information [file 41598_2020_58192_MOESM1_ESM.pdf]

Supplementary: Superconducting phase transitions in disordered NbTiN films

M. V. Burdastyh and A. Yu. Mironov
A. V. Rzhanov Institute of Semiconductor Physics SB RAS,
13 Lavrentjev Avenue, Novosibirsk, 630090 Russia and
Novosibirsk State University, 2 Pirogov Street, Novosibirsk, 630090 Russia

S. V. Postolova
A. V. Rzhanov Institute of Semiconductor Physics SB RAS,
13 Lavrentjev Avenue, Novosibirsk, 630090 Russia and
Institute for Physics of Microstructures RAS, GSP-105, Nizhny Novgorod, 603950 Russia

T. Proslier
Institut de recherches sur les lois fundamentales de l'univers,
Commissariat de l'énergie atomique et aux énergies renouvelables-Saclay, Gif-sur-Yvette, France

S. S. Ustavshikov and A. V. Antonov
Institute for Physics of Microstructures RAS, GSP-105, Nizhny Novgorod, 603950 Russia

V. M. Vinokur
Materials Science Division, Argonne National Laboratory, 9700 S. Cass Ave, Argonne, IL 60439, USA

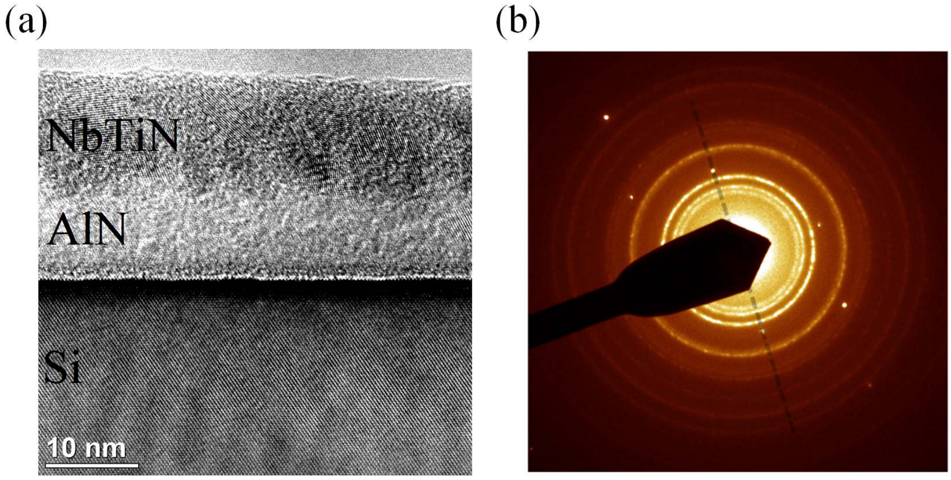

FIG. 1. **Structure of NbTiN film.** (a) Cross-section of the $S2$—1 film from High Resolution Transmission Electron Microscopy (HRTEM). (b) Electron-diffraction data of the $S2$—1 film. The rings are characteristic of polycrystalline structures; the bright spots arise from the crystalline lattice of the underlying Si substrate.

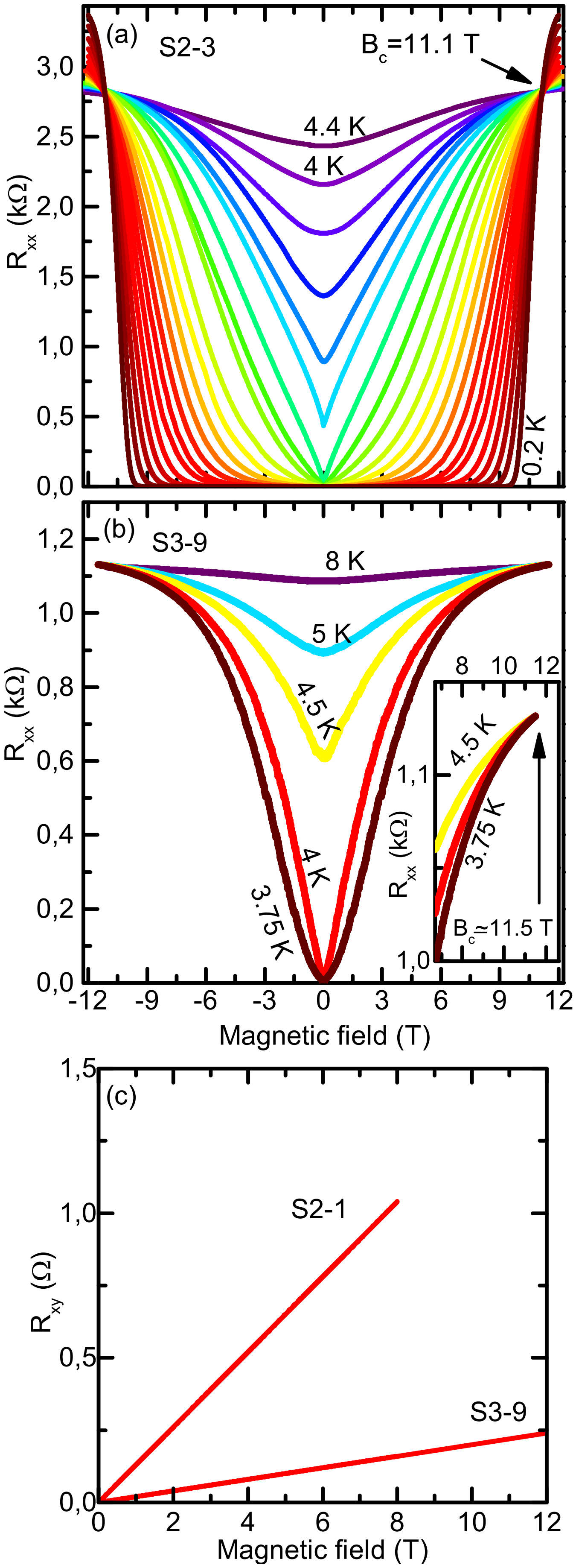

FIG. 2. **Magnetotransport** $R_{xx}(B)$ **and** $R_{xy}(B)$**.** (a) Isotherms $R_{xx}(B)$ for sample S2-2 with crossing point at $B_c = 11.1\,\mathrm{T}$ (same curves as in Fig. 2 (c) in work[4]), curves from 0.2 K to 4 K are given with 0.2 K step. (b) Isotherms $R_{xx}(B)$ for sample S9-10 imply crossing point at $B_c \gtrsim 11.5\,\mathrm{T}$. We extract $B_{c2}(0)$ from the crossing point of $R(B)$ isotherms (see SI), where $B_{c2} \approx B_c/1.04$[1]; (c) Hall resistance $R_{xy}(B)$ in normal state at $T \approx 10\,\mathrm{K}$.

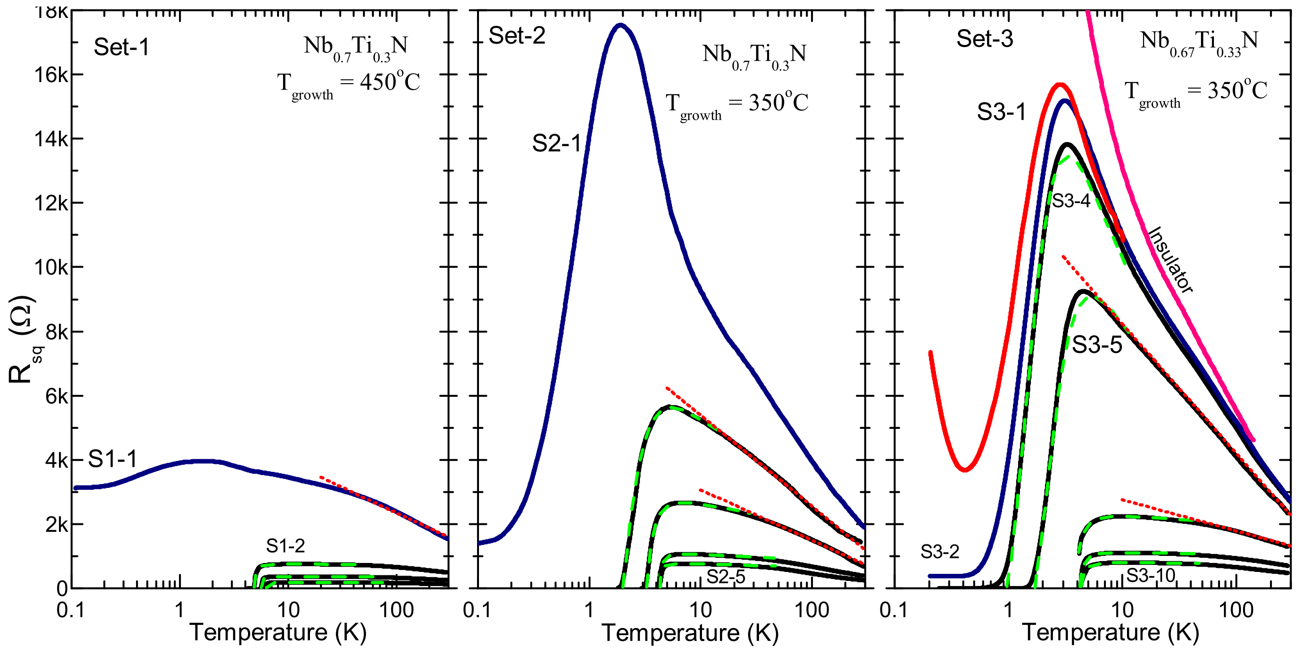

FIG. 3. $R(T)$ **in semi-log scale:** Resistance per square vs. temperature for all sets listed in table 1. Solid lines: experimental data. Dashed lines: QC fits. Insulating sample was obtained from sample S3-5 by soft plasma etching. Dotted lines show the $R \propto \ln(1/T)$ dependence, a similar behavior of the resistance was previously observed in granular systems and has not been completely explained yet[2]. Also this $R_\square \propto \ln(1/T)$ dependence differs from TiN films where the conductance depends logarithmically on temperature $G = 1/R_\square \propto \ln(T)$ (due to weak localization[3]).

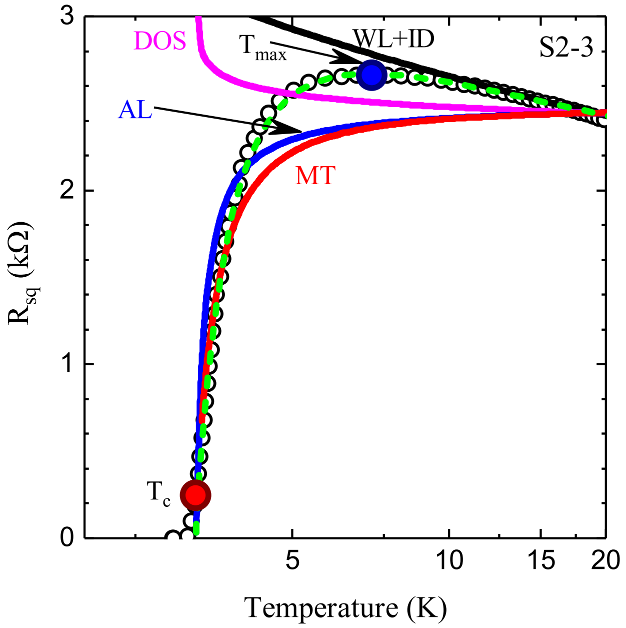

FIG. 4. **Quantum corrections to conductivity.** Resistance per square vs. temperature for film S2-3. Solid lines: fits accounting for all the quantum contributions. Dashed lines (marked as WL+ID): separate contribution of the sum of weak localization and interaction in the diffusion channel to the resistance of the sample S2—3. Dotted lines (SF): contribution of superconducting fluctuations. Bold dots denote characteristic temperatures T_c and T_{max}

FIG. 5. **BKT transition in** $R(T)$**.** Temperature dependences of the resistance per square of S1-4, S2-3, S3-10 films on logarithmic scale versus the reduced temperature (T_{BKT} values are given in table in main text). The solid lines correspond to Eq. (2) in main text.

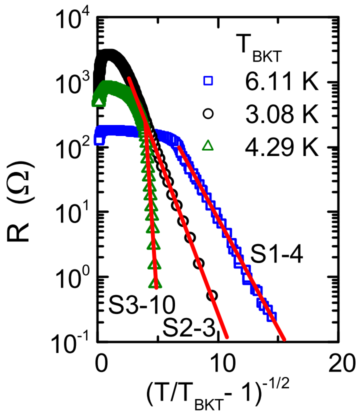

[1] Gantmakher, V. F., Ermolov, S. N., Tsydynzhapov, G. E., Zhukov, A. A., Baturina, T. I. Suppression of 2D superconductivity by the magnetic field: quantum correction vs superconductor-insulator transition. JETP Letters **77**, 424 (2003).

[2] Beloborodov, I. S., Lopatin, A. V., Vinokur, V. M. & Efetov, K. B. Granular electronic systems. Rev. Mod. Phys. **79**, 469 (2007).

[3] Baturina, T. I. et. al. Superconducting phase transitions in ultrathin TiN films. Europhys. Lett. **97**, 17012 (2012).

[4] A. Yu. Mironov, D. M. Silevitch, T. Proslier, S. V. Postolova, M. V. Burdastyh, A. K. Gutakovskii, T. F. Rosenbaum, V. M. Vinokur, T. I. Baturina. Charge Berezinskii-Kosterlitz-Thouless transition in superconducting NbTiN films. Scientific Reports **8**, 4082 (2018).
